# *Gemella haemolysans *Infection in Total Hip Arthroplasty

**DOI:** 10.1155/2012/691703

**Published:** 2012-05-09

**Authors:** Barry Rose, Parminder J. S. Jeer, Anthony J. Spriggins

**Affiliations:** ^1^Department of Orthopaedics, Queen Elizabeth The Queen Mother Hospital, East Kent Hospitals NHS Foundation Trust, St. Peters Road, Margate, Kent CT9 4AN, UK; ^2^Orthopaedic Department, SPORTSMED. SA, 32 Payneham Road, Stepney, SA 5069, Australia

## Abstract

*Gemella haemolysans* is a Gram-positive coccus and commensal of the upper respiratory tract and oral mucosa that rarely causes clinically important infections. There is only one previous report of this organism causing periprosthetic infection, in a total knee arthroplasty. We present a case of septic loosening of an uncemented total hip arthroplasty due to *G. haemolysans*, in an asplenic patient with insulin dependent diabetes mellitus. Treatment with two-stage revision has been successful at 7 years of follow-up.

## 1. Introduction

Deep late infection following total hip arthroplasty (THA) is an uncommon but devastating complication. There are numerous factors associated with such infections, including those related to both the host immunologic status and the infecting organism. The adverse effects of hyperglycaemia in diabetic patients [1] and asplenia [2] as risk factors for infection are well recognised. The organisms most commonly isolated in deep infection following THA are *Staphylococcus aureus* and *Staphylococcus epidermidis* [3, 4]. *Gemella haemolysans* is a Gram-positive bacterium and normal commensal of the upper respiratory tract and oral mucosa. It is being increasingly isolated as the causative organism in a number of infections, including meningitis, cerebral abscesses, endophthalmitis, keratitis, pneumonia, endocarditis, glomerulonephritis, and peritonitis [5–13]. It is extremely rare for this organism to cause infections of an orthopaedic nature, with only three reports of spinal infections [14–16], and a single report of infection in a total knee arthroplasty (TKA), which occurred in a patient with rheumatoid arthritis [17]. We report the first case of septic loosening in THA due to *G. haemolysans* infection 5 years following the uncomplicated primary surgical episode.

## 2. Case Report

A 40-year-old male with primary hip osteoarthritis underwent left THA in October 1997 using an uncemented prosthesis with a polyethylene bearing surface. The patient had been an insulin-dependent diabetic since 1986 and in 1994 had undergone splenectomy following complications during a Nissen fundoplication. As a result of this, he had received antipneumococcal vaccination, but despite recommendations to take a regular dose of empirical phenoxymethylpenicillin, he admitted poor compliance with this.

Prior to THA, routine urine and sputum cultures were free of infection, and his blood sugar levels revealed good diabetic control. He had been examined by a senior physician and had no cardiorespiratory problems. The THA procedure was covered with intravenous phenoxymethylpenicillin, gentamicin, and cefuroxime at induction, and these were continued to the third postoperative day, followed by resumption of oral phenoxymethylpenicillin prophylaxis. His postoperative course was unremarkable, with no wound complications. Clinical and radiographic review on a yearly basis to October 2001 was satisfactory.

At five years after surgery he presented with acute left groin pain with radiation to the thigh without history of antecedent trauma. He stated that he had felt lethargic and that his blood glucose levels had recently been elevated and difficult to control.

Examination revealed no evidence of hip wound infection and a pain-free range of hip movement. He was found to be pyrexial with a temperature of 37.9°C and had a white cell count of 16.6 × 10^9^/L, an erythrocyte sedimentation rate (ESR) of 98 mm/hr, and a C-reactive-protein of 89 mg/L (normal range <10 mg/L). His diabetic control was poor with an Hb^A1C^ percentage >11%. Subsequent urine, sputum, and blood cultures did not reveal any focus of infection, as was the case with physical examination of all other body systems. Plain radiographs of the left hip revealed no obvious signs of loosening or fracture.

A bone scan revealed a focus of hyperaemia in the region of the acetabulum in the early blood pool frames and delayed images revealed increased activity around the femoral component in Gruen zone 7. A Gallium scan revealed moderately increased isotopic activity at the superolateral aspect of the acetabular component and around the proximal femur in Gruen zones 1 and 7. The hip was aspirated in theatre revealing turbid fluid, and cultures after five days of incubation revealed *G. haemolysans*. The organism was sensitive to penicillin with a minimum inhibitory concentration of 0.06 mg/L.

An exploration of the hip joint was performed with a view to performing a two-stage revision if there was evidence of component loosening. At surgery antibiotic prophylaxis was not withheld in view of the risk of overwhelming sepsis in an asplenic patient, and high-dose intravenous benzylpenicillin was administered. The hip joint was found to be obviously infected, and pus sent for microbiological analysis along with multiple tissue specimens. The acetabular component was loose and easily removed. There was osteolysis around the proximal femur, but the femoral component was well fixed distally and required a femoral osteotomy to be removed. The hip was debrided and irrigated with hydrogen peroxide, povidone iodine, and saline impregnated with flucloxacillin at 2 g/L concentration, and a PROSTALAC (prosthesis of antibiotic-loaded acrylic cement) gentamicin femoral spacer (Depuy, Warsaw, Indiana) was inserted. All intraoperative fluid and tissue samples grew a pure isolate of *G. haemolysans* sensitive to penicillin.

Intravenous penicillin treatment was continued for six weeks at which time the patient was systemically well, with improved diabetic control and uncomplicated wound healing. The inflammatory markers had improved with an ESR of 18 mm/hr and a CRP of 17 mg/L. The second-stage revision was performed at this stage, using a long stem uncemented femoral component and an uncemented acetabular prosthesis. There were no intraoperative signs of infection and tissue samples from this procedure failed to grow any organisms. This procedure was again covered with intravenous benzylpenicillin which was continued until discharge at two weeks, followed by high-dose oral phenoxymethylpenicillin (1 g six hourly) for four weeks. At 7-year follow-up, clinical and radiographic reviews have been satisfactory, with normalisation of inflammatory markers.

## 3. Discussion

*G. haemolysans* was first described in 1938 by Thjotta and Boe as Neisseria haemolysins [18] and later allocated a separate genus by Berger, following enzymatic studies [19]. The chemical composition of the peptidoglycan of *G. haemolysans* is identical to that of Gram-positive bacteria [20]. It is a catalase negative, facultative anaerobe coccus, which resembles and has an IgA protease similar to *Streptococcus viridans* [21]. The organism may be identified by a series of biochemical reactions (API 20 Streptococcus identification system, BioMérieux, Marcy l'Etoile, France) and Gram-staining [17]. However, identification of this organism commonly presents difficulties as solely biochemical analysis may produce erroneous identification. In this case, initial characterisation was performed using the API-ZYM system (BioMérieux, Marcy l'Etoile, France) supplemented by additional biochemical testing. Definitive identification was confirmed using 16S ribosomal RNA gene sequencing [22].

*G. haemolysans* is a normal commensal of the upper respiratory tract and found in approximately 30% of healthy adults in the nasopharyngeal mucosa [23]. The reported cases of *G. haemolysans* in the literature have occurred in immunocompetent patients [5–16], although the single case of infection following TKA was in a rheumatoid patient with a history of treatment with disease modifying antirheumatic drugs [17]. This patient was successfully managed with a two-stage revision and penicillin antimicrobial chemotherapy.

There have been three case reports of *G. Morbillorum*-infected arthroplasties (two hips, one elbow) [24–26], which is closely related to *G. haemolysans*. All three were successfully managed by two-stage revision surgery. Two further cases of *G. morbillorum* septic arthritis (one hip, one knee) [27, 28] and one of spondylodiscitis with epidural abscess following an acute lumbar vertebral fracture [29] have also been described.

The onset of late deep infection in this case suggests a haematologic cause [3, 30, 31]. Although our patient did not have any history of recent dental surgery or obvious dental hygiene problems prior to presentation, the most likely explanation for this haematologic cause is a transient bacteraemia, and this is more likely in a patient with poor dental health [32, 33].

Asplenic patients are at an increased risk of infection with encapsulated bacteria. There is evidence that lifelong antibiotic prophylaxis with penicillin may reduce the risk of infection but not entirely eliminate it [34]. Our patient was poorly compliant with penicillin prophylaxis. However, *G. haemolysans* is not an encapsulated organism. As such, the asplenic status of this patient is unlikely to be of significance, unless another unidentified organism was in part responsible for the infection. Poor diabetic control is more likely to have contributed to the risk of a periprosthetic infection.

## 4. Conclusion

*G. haemolysans* is an extremely rare cause of periprosthetic infection in immunocompromised hosts. This rarity may be related to difficulties characterising it using traditional methods. Periprosthetic infection with this organism can be successfully managed with two-stage revision combined with antibiotic treatment.
